# A Mode of Compressing the Abdominal Aorta

**Published:** 1894-04

**Authors:** 


A MODE OF COMPRESSING THE ABDOMINAL AORTA.—In the January number of the *Annals of Surgery* Professor William Macewen, of Glasgow, thus describes a method that he says he has tested sufficiently to feel warranted in making it public:

As the patient lies on his back on the table, the assistant, facing the patient's feet, stands on the left side of the table in a line with the patient's umbilicus. He then places his closed right hand upon the patient's abdomen a little to the left of the middle line, the knuckles of the index finger just touching the upper border of the umbilicus, so that the whole shut hand will embrace about three inches of the distal extremity of the aorta above its bifurcation. The assistant then, standing upon his left foot, his right foot crossing his left and resting upon the toes of the right—an attitude commonly assumed by public speakers—leans upon his right hand and thereby exercises the necessary amount of pressure. With the index finger of the assistant's left hand the weight necessary for the purpose can easily be estimated by the effect produced upon the flow of blood through the common femoral at the brim of the pelvis. Whenever the flow of blood through the femorals is absolutely arrested the abdominal aorta is sufficiently controlled, and no further weight ought to be applied.

The weight exercised can be varied at will by increasing or decreasing the angle which the assistant's body makes with the floor.

As the abdominal aorta sometimes bifurcates higher than usual, before the operation is commenced a trial of the effect of the pressure at the part selected ought to be made, testing the result of pressure on both femorals. When both are equally controlled the bifurcation occurs below the point pressed on; when only one is controlled the hand requires to be placed on a more proximal part.

As there is no direct muscular effort required in maintaining the pressure further than the preservation of the equilibrium, the position can be maintained by the assistant without undue strain on his part, and without shifting his hand for at least half an hour, a time amply sufficient for the performance of most operations requiring the control of the circulation through the abdominal aorta. —*New York Medical Journal.*

